# Language Learning Variability within the Dorsal and Ventral Streams as a Cue for Compensatory Mechanisms in Aphasia Recovery

**DOI:** 10.3389/fnhum.2017.00476

**Published:** 2017-09-27

**Authors:** Diana López-Barroso, Ruth de Diego-Balaguer

**Affiliations:** ^1^Cognitive Neurology and Aphasia Unit, Cathedra ARPA of Aphasia, Centro de Investigaciones Médico-Sanitarias and Instituto de Investigación Biomédica de Málaga, University of Malaga, Malaga, Spain; ^2^Area of Psychobiology, Faculty of Psychology, University of Malaga, Malaga, Spain; ^3^Cognition and Brain Plasticity Group, Bellvitge Biomedical Research Institute (IDIBELL), L’Hospitalet de Llobregat, Barcelona, Spain; ^4^Department of Cognition, Development and Educational Psychology, University of Barcelona, Barcelona, Spain; ^5^Institució Catalana de Recerca i Estudis Avançats (ICREA), Barcelona, Spain; ^6^Institute of Neurosciences, University of Barcelona, Barcelona, Spain

**Keywords:** language learning, aphasia rehabilitation, dorsal stream, ventral stream, individual differences, brain plasticity

## Abstract

Dorsal and ventral pathways connecting perisylvian language areas have been shown to be functionally and anatomically segregated. Whereas the dorsal pathway integrates the sensory-motor information required for verbal repetition, the ventral pathway has classically been associated with semantic processes. The great individual differences characterizing language learning through life partly correlate with brain structure and function within these dorsal and ventral language networks. Variability and plasticity within these networks also underlie inter-individual differences in the recovery of linguistic abilities in aphasia. Despite the division of labor of the dorsal and ventral streams, studies in healthy individuals have shown how the interaction of them and the redundancy in the areas they connect allow for compensatory strategies in functions that are usually segregated. In this mini-review we highlight the need to examine compensatory mechanisms between streams in healthy individuals as a helpful guide to choosing the most appropriate rehabilitation strategies, using spared functions and targeting preserved compensatory networks for brain plasticity.

## Introduction

**Figure 1:**
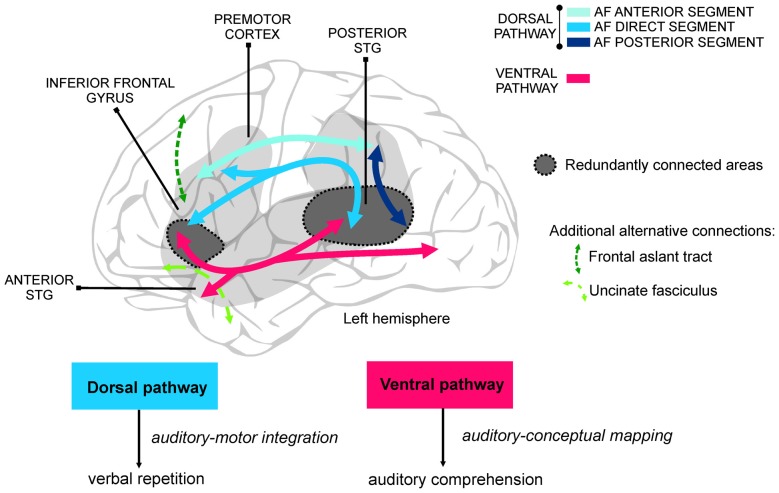
Division of labor of the ventral and dorsal pathways for language. On the top is shown a schematic illustration of the main anatomical connections of the perisylvian areas in the left hemisphere, comprising the ventral and the dorsal pathways. The perisylvian cortex, covering frontal, parietal and temporal cortices, is shaded in gray. Compensation between the dorsal and the ventral streams, normally associated with poorer performance, derives from the partial overlap of the cortical areas they connect and the extra support of additional language-related white-matter pathways (e.g., uncinate fasciculus, frontal aslant tract). Regions showing greater likelihood to be redundantly connected through the ventral and dorsal pathways are colored in dark gray. Compensation mechanisms are crucial in high difficulty language situations and after brain damage. At the bottom, the main functions of the dorsal and ventral streams are illustrated. Although not shown in the figure, the homologous areas in the right hemisphere may also play a crucial role in compensation mechanisms. AF, arcuate fasciculus; STG, superior temporal gyrus.

Language learning during adulthood is subject to huge individual differences. Recent studies using multi-modal neuroimaging have shown how this variability is reflected in the structural and functional properties of the neural network supporting different language functions (Rodríguez-Fornells et al., 2009). The language network is divided into two separate anatomical streams (Hickok and Poeppel, 2004, 2007; Friederici and Gierhan, 2013) that arise from the posterior superior temporal gyrus (see Figure 1) and appear to be specialized in complementary functions. On one hand, the *dorsal stream* projects towards the inferior parietal and posterior frontal lobe (inferior frontal gyrus and premotor cortex) through the arcuate fasciculus (AF; Wernicke, 1874; Catani et al., 2005; Saur et al., 2008) and is in charge of the translation of sensory/acoustic speech signals into motor-articulatory representations (auditory-motor integration) required for verbal repetition. On the other hand, the *ventral stream* links the superior and middle temporal gyri, the inferior parietal lobe, and the occipital lobe with the inferior frontal gyrus through the inferior fronto-occipital fasciculus (Martino et al., 2010; also called extreme capsule fiber system, see Friederici and Gierhan, 2013). Additionally, the most anterior parts of the inferior frontal gyrus and the frontal operculum are connected to the anterior temporal lobe through the uncinate fasciculus (Friederici and Gierhan, 2013). The ventral stream is mainly involved in the mapping of sensory/auditory speech signals into conceptual and semantic representations for speech comprehension.

As occurs in normal language function, this division of labor is reflected in the language deficits associated with damage to one of these streams. After a stroke, especially in the left perisylvian areas, problems can arise in the production and/or comprehension of speech (aphasia). In particular, lesions in the ventral pathway are mostly associated with auditory comprehension deficits (Kümmerer et al., 2013) whereas lesions of the dorsal pathway are classically associated with repetition deficits (conduction aphasia; Wernicke, 1874; Kümmerer et al., 2013). The plastic changes deriving from these lesions are nevertheless highly variable. They depend in part on the size of the lesion and the prior language network organization (more or less left-lateralized; Berthier et al., 2012). More importantly, they depend also on plasticity within the language network in relation to the spontaneous perilesional recovery and the recruitment of the homologous right hemispheric frontal areas (Heiss et al., 1999; Berthier et al., 2012).

The study of virtual lesions (i.e., using transcranial magnetic stimulation, TMS) and of the individual differences in healthy subjects learning new words point also to the possible role of mutual compensation between dorsal and ventral language streams to explain part of the variability in language recovery in aphasia. For example, the ability to repeat sounds (words or pseudo-words) represents a major criterion in classifying aphasia syndromes (Kertesz, 1979). Interestingly repetition ability, which preferentially relies on auditory-motor integration through the dorsal pathway (Hickok and Poeppel, 2007; Saur et al., 2008; Rauschecker and Scott, 2009), is also crucial when healthy adults learn new phonological word forms (López-Barroso et al., 2013). However, the ventral pathway seems to play the leading role when the dorsal AF is not available, although this results in suboptimal performance (López-Barroso et al., 2011). Thus, it seems that in spite of the functional specialization of the dorsal and ventral streams, certain compensatory functions occur between them, and this might be possible thanks to the partial redundancy existing in the connectivity between language areas (Figure 1).

In this mini-review, our aim is to highlight how the evidence from inter-individual differences in language learning performance and their relation with dorsal and ventral pathways of speech processing in the healthy brain can help us to understand compensatory mechanisms in aphasia recovery and help tailor optimal neurorehabilitation strategies. We will focus mainly on phonological word learning since repetition is tightly linked to this capacity and it is a common impairment in aphasia. Therefore, we will present studies that have looked at individual differences in the learning of new words and how this ability relates to auditory-motor integration through the dorsal language network. We will then review evidence supporting the mutual compensatory role between dorsal and ventral language streams and we will link them with compensatory behaviors observed in aphasia.

## Individual Differences in Word Learning

### Individual Differences in Word Learning in Relation to the Dorsal Pathway

Language learning is a multifaceted process that requires mastering different components such as words, grammar and speech sounds. As we will see in the following section, the learning of phonological word forms is supported by the rehearsal component of phonological working memory, which involves a covert repetition process (Baddeley et al., 1998). Given the importance of repetition in aphasia we will focus here on the inter-individual differences in the word learning process. In this respect, it is important to understand the dorsal stream/ventral stream dissociation in terms of both anatomy and its related language functions in order to tease apart the different aspects that need to be mastered for new word learning, such as the creation of a memory trace for the phonological word form and its association with the semantic content. In a clinical setting, this division of labor may allow us to gain further insight into the spared and possible alternative networks that can be targeted to improve performance. In addition, it is important to note that different cognitive functions including attention, cognitive control and working memory are featured in the dorsal stream (Wise et al., 2001; Corbetta and Shulman, 2002; Buchsbaum and D’Esposito, 2008; Salmi et al., 2009). Indeed, these cognitive functions have also been described as being important for the learning of new words (Baddeley, 2003; de Diego-Balaguer et al., 2007; Hickok and Poeppel, 2007; Rodríguez-Fornells et al., 2009; Schulze et al., 2012) and they are individual cognitive predictors for aphasia recovery as well (Brownsett et al., 2014).

The brain areas involved in these cognitive functions within the dorsal stream may be separated into different functional subnetworks which work in coordination during learning. Healthy individuals exposed to an artificial language (pre-segmented word forms in a speech stream with no semantic content) can progressively create memory traces of these words (de Diego-Balaguer et al., 2007; Shtyrov et al., 2010; Shtyrov, 2012). A functional magnetic resonance imaging (fMRI) study in which independent component analysis (ICA; Calhoun et al., 2008) was performed on the blood-oxygen-level dependent (BOLD) signal showed different sub-networks within the dorsal stream to be engaged in parallel during word-learning periods and disengaged at rest (López-Barroso et al., 2015). This suggests that these independent subnetworks may be related to different functions that are coordinated in the process of learning. Moreover, a fronto-temporal network within the ventral stream was also implicated in task performance. Although classically related to processing of semantic comprehension (Saur et al., 2008), this ventral network also seemed to be engaged even when no semantic information was present in the task, consistent with its role in the processing of local dependencies (Friederici et al., 2006) and auditory object recognition (Rauschecker and Scott, 2009; Bornkessel-Schlesewsky et al., 2015). This supports the imbricate relationship between the dorsal and ventral pathways despite their specialized roles, thus highlighting how these pathways work synergistically to achieve common goals in some cognitive operations (Rolheiser et al., 2011). This evidence is particularly important for understanding the role of neurorehabilitation in the remodeling of neural networks, in which the use of one pathway during behavioral training can have direct effects on the other. Nevertheless, it is important to note that although all these networks were significantly engaged as a function of the task, only the strength of connectivity of the auditory-premotor network correlated positively with word learning performance (López-Barroso et al., 2015). This not only suggests cooperation between the two pathways but also emphasizes the specific importance of audio-motor coupling in successful learning of word forms.

In this respect, a complementary study showed convergent results when connectivity analysis was restricted to the dorsal stream (López-Barroso et al., 2013). The strength of functional connectivity between the premotor and superior temporal regions in the left hemisphere significantly correlated with individual differences in word form learning performance. The importance of the close direct connection between these regions in successful learning was also reflected by the association between word learning performance and the structural connectivity of the AF connecting these same areas (López-Barroso et al., 2013). The crucial role of the AF in the rapid cross-interaction between the left frontal and temporal areas suggests that functions depending on this white matter bundle play an important role in the initial stages of language learning when memory traces of word forms are created (Schulze et al., 2012). The binding between acoustic and articulatory representations through the AF may facilitate the creation of the motor code required to articulate the sequence as well as the maintenance of this code in an active state through phonological working memory (Jacquemot and Scott, 2006; Buchsbaum and D’Esposito, 2008). Indeed aphasic patients with spared frontal areas and dorsal pathway are able to learn new words from fluent speech (Peñaloza et al., 2015).

### Individual Differences in Word Learning in Relation to Phonological Working Memory

In terms of the underlying function facilitated by this dorsal stream, there is extensive evidence linking the phonological working memory capacity with the acquisition of new vocabulary. This has been shown in children (Baddeley et al., 1998; Baddeley, 2003) and in adult learning of a second language (Papagno et al., 1991), as well as in some aspects of syntax (Ellis and Sinclair, 1996). Phonological working memory is composed of the phonological store, which can hold limited memory traces for a few seconds before they decay, and the articulatory rehearsal. The rehearsal process refreshes these memory traces by retrieval and re-articulation (i.e., inner speech; Baddeley and Hitch, 1974; Baddeley et al., 1998; Baddeley, 2000). The link between phonological working memory and new word learning operates only when new phonological forms need to be repeated, but it does not seem to apply in the learning of associations of already known words to new meanings (Gathercole and Baddeley, 1990). This aspect is of interest when thinking about rehabilitation procedures in aphasia, since aphasic syndromes with impaired verbal repetition (conduction aphasia, Broca’s aphasia, Wernicke’s aphasia) are more likely to show associated working memory alterations. More precisely, it has been shown that the rehearsal subcomponent of working memory plays an important role in word learning from fluent speech in adults (López-Barroso et al., 2011). When the rehearsal mechanism is artificially interfered by articulatory suppression, the information that lies in the short-term phonological store rapidly decays, inducing drastic effects on learning performance (López-Barroso et al., 2011). The articulatory rehearsal is a repetition mechanism that depends on the same brain mechanisms that underlie speech production (Buchsbaum and D’Esposito, 2008), thus relying on AF connectivity. Indeed, this mapping fits well with the specific relationship of the rehearsal component with individual differences in word learning since the long segment of the AF connects the posterior part of the superior temporal and the inferior frontal gyri and premotor cortex, these two latter areas being associated with the rehearsal capacity (Paulesu et al., 1993; Awh et al., 1996; Jonides et al., 1998).

## Compensatory Functions of Language-Related White Matter Tracts

Despite the functional specialization of the dorsal stream in auditory-motor integration and the ventral stream in semantic processing, the language system is highly flexible. Recent models have proposed that the ventral stream is actually involved in the identification of auditory objects included in speech. This identification happens in a hierarchical manner from more posterior regions identifying phonemes and syllables to progressively more anterior regions identifying words and phrases and overall sentence comprehension (Rauschecker and Scott, 2009; Bornkessel-Schlesewsky et al., 2015). Thus, according to these theoretical elaborations, the ventral stream plays a role not only in semantic processing but also in more formal identification of auditory objects. This idea is consistent with the engagement of the ventral together with the dorsal stream during the learning of new word forms from a speech stream even when semantic information is not present, since it requires the creation and identification of memory traces of word forms (López-Barroso et al., 2015). Thus, in spite of the division of labor of the language streams, the additional engagement of the ventral and other dorsal subnetworks may represent a compensatory mechanism or additional support for the achievement of a complex task. This possibility is supported by the fact that under rehearsal blockage interfering with the use of the dorsal audio-motor communication, healthy individuals are still able to learn new word forms, albeit with a reduced performance (López-Barroso et al., 2011). In this condition, individual differences in language learning were associated with microstructural differences in the extreme capsule, consistent with the anatomical trajectory of the ventral stream. Thus, the ventral pathway may act as an alternative connection when the dorsal pathway is unavailable. This may shed light on some aspects of aphasia rehabilitation, especially when dorsal lesions interfering with repetition ability exist. For example, presenting words (words that were lost after brain damage) submerged in a rich semantic context while requiring the patient to repeat them may potentiate the use of the ventral pathway for repetition, thus promoting “re-learning” success.

Compensatory effects between streams can also be observed in the case of virtual lesions through the use of TMS, which allows the study of the behavioral effects resulting from the transitory interference of a given brain region. Hartwigsen (2016) and Hartwigsen et al. (2016) showed that the ventral stream in healthy subjects was more resilient to semantic interference than the dorsal stream to disruption of phonological processing. Interestingly, on inhibition of the frontal or parietal cortices linked by the ventral stream, the non-stimulated site compensated for performance. Only concurrent stimulation of both the frontal and parietal sites led to increased reaction times in semantic processing. In contrast, focal stimulation of either the frontal or parietal cortices linked by the dorsal stream led to significant interference effects in a phonological task. Additionally, fMRI obtained under the effects of TMS indicated that dorsal regions in the inferior parietal lobe (supramarginal gyrus) also showed increased compensatory activation when the semantic system was interfered with by brain stimulation (Hartwigsen, 2016).

Thus, although there are preferred pathways to efficiently connect two or more cortical areas, the existence of alternative pathways could partially ensure the functionality of the system in cases in which the main pathway is occupied with another task, is still immature, or is dysfunctional (see Figure 1; Nozari and Dell, 2013). In particular, the ventral stream could play a supporting role during processes that are normally associated with the dorsal stream. This compensatory role is likely performed through the use of cognitive strategies (possibly less efficient) different from those used by the dorsal stream, taking into account that the areas connected through these streams and their functionality are different.

The compensatory mechanism observed through the study of individual differences in healthy participants is similar to what is observed in cases of dorsal lesions. After anterior temporal lobe resections epileptic patients show improved language recovery when structural plasticity is observed in the ipsilateral ventro-medial language network (Yogarajah et al., 2010). In a similar vein, Yeatman and Feldman (2013) reported the case of an adolescent girl with bilateral periventricular perinatal leukomalacia leading to the complete absence of the long and anterior segments of the AF. Surprisingly, language abilities (including repetition) were within normal limits as a result of increased ventral stream connectivity. Nevertheless, patient response times were slow, a finding that is in line with a fairly good compensation via the ventral stream, which was not as efficient as the preferential pathway (dorsal stream).

The compensation between streams derives from their highly interactive nature, allowing some level of redundancy in the pathways within the language system (Figure 1). Saur et al. (2010) suggested that although the superior temporal region mainly interacts with the premotor cortex via the AF (Frey et al., 2008; Saur et al., 2008), these regions complementarily interact through the extreme capsule system and the frontal operculum (which is richly connected with the premotor region, Schmahmann et al., 2007). It has been hypothesized that this alternative via may be important for the control of the dorsal sensory-motor loop during speech perception. Rolheiser et al. (2011) studied the main pathways and functional activations carrying distinct aspects of language processing in stroke patients with lesions of different locations and sizes. They found that although some functions depended on specific tracts, such as phonological processing in the dorsal stream and semantic processing in the ventral stream, functions such as syntax and morphological processing depended upon the activity of both pathways. Indeed, the development of the dorsal and ventral pathways during infancy does not run in parallel. While the ventral pathway is present at birth, the dorsal pathway is not yet detectable (Perani et al., 2011). Further, 7-year-old children, who still have an immature dorsal pathway, rely more on the ventral pathway during sentence comprehension, a task which in adults relies on dorsal stream activity (Brauer et al., 2011). However, for functions in which there is a preferred pathway, the alternative pathway might not allow for the same level of proficiency as the optimal pathway either in patients (Rauschecker et al., 2009; Yeatman and Feldman, 2013) or in healthy participants (López-Barroso et al., 2011).

### Behavioral Symptoms in Aphasia Resulting from Compensatory Plastic Mechanisms between Streams

The compensatory effects between the phonological and semantic systems reported in healthy subjects are consistent with what has recently been observed in aphasia (Berthier et al., 2017b; López-Barroso et al., 2017). A clear example of a compensatory process of the ventral stream after dorsal pathway damage is a characteristic behavioral symptom of conduction aphasia (fluent aphasia with preserved comprehension and impaired repetition; Kohn, 1992), *conduite d’approche (CdA)*. CdA is a progressive phonological approximation to a target word which reflects an attempt to self-repair production errors (Shallice and Warrington, 1977). Computational implementations of aphasia suggest that *CdA* represents an example of symptomatic suboptimal verbal behavior due to compensatory processes carried out by the ventral stream when the dorsal stream is not working properly (Ueno and Lambon Ralph, 2013). In other words, *CdA* reflects the attempt, albeit not always successful, of the intact semantic ventral pathway to clean-up the poor performance in speech production tasks (repetition, naming) of the damaged dorsal pathway (Ueno and Lambon Ralph, 2013). It is interesting to note that the reverse pattern can also be observed in the case of damage to the ventral stream. The attempt of the dorsal stream to compensate for the deficit might result in mitigated echolalia (ME). Echolalia is defined as the repetition of words and/or utterances spoken by another person (Berthier et al., 2017a), and ME is a mild form of echolalia characterized by the introduction of changes (intonation or verbal content) as an echoed emission, in general for communicative purposes (Pick, 1924; Berthier et al., 2017a,b; López-Barroso et al., 2017). ME usually occurs in fluent aphasia due to lesions of the ventral stream, which typically disrupt auditory comprehension at the word and sentence levels (Berthier et al., 2017b) but can also be observed in nonfluent aphasias (e.g., Broca’s aphasia; Hadano et al., 1998; López-Barroso et al., 2017). The main compensatory function of ME is to resolve impaired access to the semantic system due to ventral damage (Berthier et al., 2017b). It seems that through repetition of the just-heard verbal message (a function relying on the dorsal pathway), there is an increase in the likelihood of semantic system activation and access, eventually favoring auditory comprehension.

## Conclusion

In summary, the study of individual differences in healthy subjects can help to identify the networks involved in compensatory or boosting strategies in language learning abilities. Ventral and dorsal streams, despite their specializations, work synergistically showing compensatory roles. Knowledge of the functions preferentially associated with each of these streams will allow for the adoption of model-based neurorehabilitation programs to optimize recovery from aphasia by strengthening the specific functions of these compensatory networks in both cerebral hemispheres, potentiated with different intervention strategies (Berthier and Pulvermüller, 2011). In addition, studying the properties of anatomical and functional connectivity of the undamaged dorsal and ventral streams at the time of brain damage could be useful to predict the success of rehabilitation strategies based on intra- and inter-hemispherical compensatory mechanisms (Lunven et al., 2015).

## Author Contributions

DL-B and RD-B contributed intellectually to this work, drafted the article and revised it.

## Conflict of Interest Statement

The authors declare that the research was conducted in the absence of any commercial or financial relationships that could be construed as a potential conflict of interest.
